# Elucidating the therapeutic mechanisms of quercetin in hepatic fibrosis: an integrated metabolomic and transcriptomic analysis

**DOI:** 10.3389/fnut.2026.1864960

**Published:** 2026-07-02

**Authors:** Ruiqi Zhao, Yaxin Li, Tianfu Guo, Danyang Liu, Shufang Niu, Donghua Zheng, Jun Qi, Jianfang Sun, Feng Bai, Zhenwang Wang, Wanfu Bai

**Affiliations:** 1 Baotou Medical College, Baotou, Inner Mongolia, China; 2 The First Affiliated Hospital of Baotou Medical College, Baotou, China; 3 Institute of Bioactive Substance and Function of Mongolian Medicine and Chinese Materia Medica, Baotou Medical College, Baotou, China

**Keywords:** Chinese medicine, hepatic fibrosis, metabolomics, quercetin, transcriptomics

## Abstract

**Background:**

Combined metabolomics and transcriptomics analyses were performed to elucidate metabolites related to quercetin (Que) treatment in rats with CCl₄-induced hepatic fibrosis (HF).

**Methods:**

Histopathology, hepatic inflammatory and fibrosis-related markers, and quantitative real-time reverse transcription polymerase chain reaction (qRT-PCR) were used to profile fibrosis-associated changes.

**Results:**

QUE treatment was associated with altered whole-liver expression of lipid metabolism genes (Cpt1a, Acadm, Hadh, and Scd). Nevertheless, because the analyzed datasets were derived from the whole liver, these changes could not be mapped to their cellular source. Correspondingly, QUE treatment was associated with changes in arachidonic acid, hydroxyacyl-coenzyme A (CoA), β-hydroxybutyrate, and leukotriene A4. The aforementioned metabolites were enriched in fatty acid oxidation (FAO), arachidonic acid metabolism, ketone body biosynthesis, endoplasmic reticulum stress, and retinol metabolism.

**Conclusion:**

In summary, QUE treatment was associated with attenuation of hepatic fibrosis and lipid–energy metabolism-related changes. Due to the inability of bulk liver transcriptomic analyses to distinguish hepatocyte-derived changes from hepatic stellate cell (HSC)-derived changes, the restoration of Cpt1a should be interpreted as a whole-liver metabolic association rather than cell-specific antifibrotic evidence until further studies can validate this association.

## Graphical abstract

**Figure:**
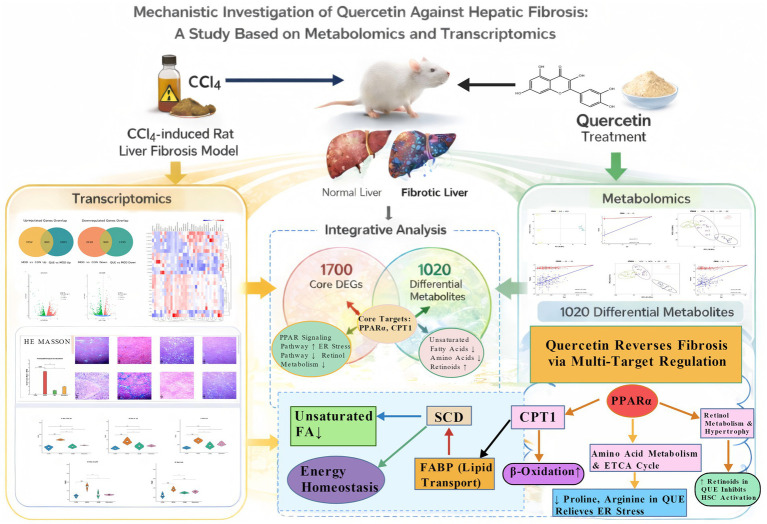

## 1 Introduction

Hepatic fibrosis (HF) is a pathological condition that develops progressively as a result of chronic liver injury from various factors, leading to sustained hepatocyte damage, a chronic inflammatory response, and excessive accumulation of extracellular matrix (ECM) components within liver tissue (1). Common etiologies include chronic viral hepatitis, alcoholic and non-alcoholic fatty liver disease, autoimmune liver disorders, and parasitic infections (2). This condition can disrupt in normal liver architecture and alter hepatic hemodynamics, potentially progressing to liver cirrhosis and hepatocellular carcinoma (HCC), both of which pose significant threats to patient survival and health (3). From a clinical perspective, no pharmacological agents are currently available to reverse HF. Existing therapeutic strategies focus on eliminating causative factors and mitigating inflammatory and fibrogenic processes (4). For instance, nucleos(t)ide analogs can suppress hepatitis B virus (HBV) replication, thereby reducing inflammation and slowing fibrosis progression (5). Antioxidant and anti-inflammatory compounds, such as silymarin, have been shown to foster a beneficial environment within the liver by inhibiting the formation of reactive oxygen species (ROS) and reducing hepatocyte apoptosis (6). Nevertheless, the efficacy of existing treatments remains constrained, with prolonged use of certain therapies is associated with hepatotoxicity, nephrotoxicity, and drug resistance, which adversely affect treatment adherence and outcomes (7).

Quercetin (Que), a naturally occurring flavonoid found in fruits, vegetables, and traditional medicinal herbs, exhibits a broad spectrum of biological activities, including antioxidant, anti-inflammatory, immunomodulatory, and antifibrotic effects (8). Quercetin and other flavonoids have been extensively studied in both traditional medicine and modern pharmacology for their potential to prevent and treat cardiovascular diseases, metabolic disorders, and chronic inflammatory conditions (9–11). Recent evidence increasingly suggests that quercetin exerts a significant protective effect against hepatic fibrosis and can inhibit the progression of fibrosis induced by various etiologies, including chronic viral hepatitis, alcoholic liver disease, and other liver disorders.

Previous investigations conducted in this laboratory have demonstrated that quercetin markedly ameliorates pathological alterations in the livers of rats subjected to CCl₄-induced hepatic fibrosis, concurrently reducing serum transaminase levels and hepatic hydroxyproline content. These findings suggest that quercetin may be a therapeutic agent for preventing or reversing the fibrotic process. It has been reported that quercetin improved pathological changes observed in experimental models of liver fibrosis and decreased serum transaminase levels and liver hydroxyproline (12). These results suggest hepatoprotective and antifibrotic activities of quercetin. Therefore, we administered a 50 mg/kg dose of quercetin, based on previously published experimental studies demonstrating the beneficial effects of quercetin in models of liver injury and fibrosis. Mechanistically, quercetin is hypothesized to exert its effects by inhibiting the transforming growth factor (TGF)-β1/Smad signaling pathway, suppressing expression of alpha-smooth muscle actin (α-SMA) and collagen I, reducing ROS production, and enhancing antioxidant defense through activation of the Nrf2/HO-1 pathway (13). Collectively, these results underscore the significant antifibrotic properties of quercetin, suggesting its potential utility for the prevention and treatment of hepatic fibrosis.

Despite existing research, the *in vivo* molecular mechanisms of quercetin remain largely undefined, particularly regarding its variable effects and molecular targets across different etiologies and disease stages (14–16). Advances in high-throughput sequencing and multi-omics approaches, such as metabolomics and proteomics, provide robust tools for exploring quercetin’s multi-target mechanisms. High-throughput sequencing identifies differentially expressed genes (DEGs) associated with hepatic fibrosis following quercetin treatment (17), while metabolomics and proteomics elucidate its regulatory effects on amino acid and fatty acid metabolism, as well as redox homeostasis. These methods also highlight key proteins and signaling networks that mediate quercetin’s antifibrotic effects (18, 19). Multi-omics analysis is instrumental in elucidating quercetin’s molecular regulatory mechanisms and identifying novel therapeutic biomarkers and drug targets (20).

In this study, we used an integrated transcriptomic and metabolomic approach to investigate molecular alterations associated with quercetin treatment in CCl₄-induced hepatic fibrosis. Our objective was to evaluate whether quercetin inhibits fibrosis progression by modulating multiple signaling pathways associated with fatty acid metabolism, energy homeostasis, and inflammatory responses, thereby delaying or preventing fibrosis progression. This investigation not only deepens our understanding of quercetin’s pharmacological properties but also offers novel insights and methodologies for using natural products as antifibrotic agents, thereby establishing a robust foundation for the future clinical applications.

## 2 Materials and methods

### 2.1 Materials and reagents

Quercetin, with a purity exceeding 98%, was obtained from Tianjin Beifang Tianyi Chemical Reagent Factory (Tianjin, China). Carbon tetrachloride (CCl₄; Batch No.: 20091217) was obtained from the same supplier. Extra Virgin Olive Oil (Batch No. 20150123) was sourced from Shandong Luhua Group Co., Ltd. (Shandong, China). Ultrapure water was supplied by Sigma Aldrich (USA), and formic acid (Chromatography Grade) was obtained from Dikma Technologies (USA).

### 2.2 Animals and ethical considerations

A total of 24 specific pathogen-free (SPF) male, 4-week-old Sprague–Dawley (SD) rats, weighing 170–200 g (*n* = 24), were obtained from the Department of Laboratory Animal Science at Peking University Health Science Center [License No. SCXK (Beijing) 2017-0005]. The rats were maintained under controlled environmental conditions, with temperatures set at 23–25 °C and humidity maintained at 50 ± 5%. They had ad libitum access to food and water. All experimental procedures were reviewed and approved by the Medical Ethics Committee of Baotou Medical College, Inner Mongolia University of Science and Technology (Approval No. 2022-961), and were conducted in accordance with international standards for the care and use of laboratory animals to minimize pain and distress.

### 2.3 Hepatic fibrosis model development and medicinal administration

After a 1-week acclimation period, the animals were randomly assigned to four groups (*n* = 6 per group): control (CON) group, model (MOD) group, quercetin-treatment (QUE) group, and silymarin positive-control group (Silymarin). Hepatic fibrosis was induced in all groups except the CON group by subcutaneous injection of 40% carbon tetrachloride (CCl₄) dissolved in olive oil into the abdominal region. The initial dose of CCl₄ was 5 mL/kg on the first day of model induction, followed by a maintenance dose of 3 mL/kg every 2 weeks for a total modeling period of 8 weeks (21). From the first day of model induction, animals in the CON and MOD groups received daily oral administration of a normal saline volume equivalent. Concurrently, animals in the QUE group were administered an oral quercetin solution at a dosage of 50 mg/kg/day, while those in the silymarin group received an oral silybin solution at a dosage of 200 mg/kg/day, both for a duration of 8 weeks. The dose of quercetin (50 mg/kg/day) was selected based on previously published data demonstrating hepatoprotective and antifibrotic effects of quercetin in experimental models of liver injury (12). As our study was designed to perform an exploratory integrated transcriptomic and metabolomic analysis, only one dose of quercetin was tested. Thus, this study was neither designed nor powered to define a dose–response relationship or determine the optimal therapeutic dose of quercetin. Silymarin/silybin was used as a pharmacological positive-control reference solely to demonstrate the responsiveness of our CCl₄-induced fibrosis model. Although we used a dose that has been previously reported in experimental models of liver injury (200 mg/kg/day), we acknowledge that it is higher than the silymarin doses used in some studies. Thus, the Silymarin group was not designed for direct dose-equivalent comparisons of efficacy with quercetin. The physical health and body weight of animals were monitored and recorded daily throughout the study.

### 2.4 Sample collection and processing

A total of 24 h following the final administration, the animals were euthanized via intraperitoneal injection of sodium pentobarbital at a dosage of 150 mg/kg. Immediately after euthanasia, the livers were excised and examined for gross morphological abnormalities, such as changes in color, texture, and surface characteristics. Liver tissue sections were fixed in 4% paraformaldehyde for subsequent histopathological analysis. The remaining liver tissue was rapidly frozen in liquid nitrogen and stored at −80 °C for the future analytical assessments.

### 2.5 Analysis of pro-inflammatory cytokines and fibrosis-associated markers in liver tissue

Approximately 0.1 g of freshly harvested liver tissue from each rat was homogenized in approximately 9 volumes of cold normal saline and then centrifuged to collect the supernatant. The concentrations of pro-inflammatory cytokines and fibrosis-associated proteins were measured using commercially available Enzyme-Linked Immunosorbent Assay (ELISA) kits, following the manufacturers’ instructions, and quantified using an automated microplate reader. The pro-inflammatory cytokines and fibrosis-associated markers analyzed included interleukin (IL)-1β, TGF-β1, hyaluronic acid (HA), laminin (LN), and collagen type IV (Col IV). These concentrations were quantitatively assessed to evaluate the potential of quercetin to modulate the inflammatory response and collagen deposition associated with hepatic fibrosis progression.

### 2.6 Histopathological evaluation of liver tissue

Histopathological examination of liver tissue was performed using hematoxylin and eosin (H&E) staining and Masson’s Trichrome staining kits. Liver tissue sections, embedded in paraffin and sectioned at 3–4 μm, were stained using an H&E staining kit (Batch No.: 20191231, Beijing Baiolaibo Technology Co., Ltd., China). Additional deparaffinized liver sections were stained using a Masson’s Trichrome staining kit (Batch No.: 20191025). Histopathological changes were examined at 100 × magnification under a light microscope (CX31, Olympus, Japan). Liver fibrosis was assessed using the Ishak Scoring System, which is defined as follows:

0 = no fibrosis (normal liver);

1 = expansion of portal tracts with fibrous tissue;

2 = expansion of portal tracts with fibrous tissue and rare septa;

3 = rare septal bridging;

4 = bridging fibrosis with significant portal-to-portal bridging;

5 = architectural distortion with incomplete cirrhosis;

6 = established cirrhosis with pseudo-lobule formation.

### 2.7 Transcriptomic analysis

The liver fibrosis model used in this study was developed by administering carbon tetrachloride (CCl₄) to male Sprague–Dawley rats to induce hepatic fibrosis, followed by treatment with quercetin for a specified duration. Liver tissue samples were collected from the different groups after treatment for histological examination and molecular analysis. Total RNA was isolated from liver tissue samples using TRIzol reagent according to the manufacturer’s instructions. The quality of the RNA was evaluated by assessing degradation and contamination using 1% agarose gel electrophoresis. The RNA’s purity was further assessed by measuring its concentration with a NanoDrop spectrophotometer, and its integrity was determined by measuring the RNA Integrity Number (RIN) using an Agilent Bioanalyzer 2100 system. A subset of the RNA samples underwent enrichment for poly-A+ mRNA using poly-T magnetic beads, followed by conversion into complementary DNA (cDNA) through reverse transcription. These cDNA molecules were subsequently assembled into sequencing libraries. The cDNA libraries were sequenced on an Illumina platform using Next-Generation Sequencing (NGS) technology to produce raw sequence data in FASTQ format. Raw reads generated from NGS experiments often contain sequencing artifacts, such as adaptors that remain attached to the ends of the reads after sequencing. Consequently, raw reads are typically subjected to quality-control (QC) filtering prior to downstream bioinformatics analysis. In this study, the quality-control filtering process was conducted using fastp (version 0.19.7) tool to eliminate contaminant sequences, such as adapters, and low-quality reads. Specifically, reads with more than 10% ambiguous bases (N) and those with over 50% of bases having a Phred score (Qphred) of 20 or lower were removed.

This filtering step resulted in the acquisition of high-quality, clean reads devoid of contaminants and sequencing artifacts. Subsequently, these high-quality clean reads were aligned to the reference genome using HISAT2 to determine their origins. After determining the genomic locations of the clean reads, the number of reads mapping to each genomic locus was counted to generate gene-level raw counts. These counts served as input for differential expression analysis, which was performed using the DESeq2 R package. DESeq2 R package employs a negative binomial distribution model to assess the differential expression of genes between the two experimental groups under comparison. Furthermore, the DESeq2 R package conducts internal normalization of raw counts to adjust for differences in sequencing depth between the two experimental groups being compared. DEGs were defined with the DESeq2 R package using adjusted *p*-value [Benjamini–Hochberg false discovery rate (FDR)] < 0.05 and absolute log₂fold change ≥1. We applied a multiple-testing correction to limit the false-positive rate inherent in genome-wide transcriptomic analysis. To facilitate the visualization of differential expression analysis results and to cluster genes by expression level, gene expression data were converted to transcripts per million (TPM) values. These TPM values were subsequently used to generate volcano plots, illustrating the relationship between *p*-value and log_2_-fold change (FC). Correlation heatmaps were also constructed to illustrate the relationships among differentially expressed genes. Additionally, Gene Ontology (GO) and Kyoto Encyclopedia of Genes and Genomes (KEGG) pathway enrichment analyses were conducted using the OmicStudio cloud platform^1^ or the appropriate R packages. TheGene Ontology (GO) and Kyoto Encyclopedia of Genes and Genomes (KEGG) pathway enrichment analyses identified gene sets that were differentially expressed and functionally related. It is crucial to note that Transcripts Per Million (TPM) values were used for all visualizations and clustering analyses. In contrast, differential expression analysis was conducted exclusively using gene-level raw counts with the DESeq2 R package. Consequently, raw counts and TPM values should be interpreted separately and with caution.

### 2.8 UPLC-Q-TOF/MS-based metabolomic analysis

To investigate changes in the serum metabolomic profile following quercetin treatment, we employed ultra-performance liquid chromatography coupled with quadrupole time-of-flight mass spectrometry (UPLC-Q-TOF/MS). Prior to conducting supervised multivariate statistical analysis, unsupervised principal component analysis (PCA) was performed to visualize global metabolic patterns in the serum samples using MetaboAnalyst 6.0. This PCA was instrumental in visualizing overarching metabolic patterns and evaluating sample clustering. Using EZinfo 3.0 (Waters), we conducted a supervised partial least squares-discriminant analysis (PLS-DA) to assess the degree of separation among serum metabolomic profiles across treatment groups. PLS-DA, a form of supervised multivariate statistical analysis, uses the sample class labels (i.e., treatment groups) to pinpoint variables that significantly contribute to class differentiation. Beyond assessing the separation of serum metabolomic profiles, we also evaluated the PLS-DA model fit using permutation tests, facilitated by the OmicStudio cloud platform.^2^ These permutation tests allowed us to determine whether the model fit was due to random chance or reflected genuine differences in serum metabolomic profiles across the treatment groups. Differential metabolites were defined as those in serum samples with variable importance in projection (VIP) ≥ 2, *p*-value <0.05, and log_2_-FC ≥ 1. We used a relatively strict VIP threshold to reduce potential false-positives and prioritize metabolites with higher discriminatory power. Subsequently, Venn diagrams were constructed to identify the overlap of differential metabolites between the CON vs. MOD and MOD vs. treatment groups, to determine whether any metabolites were common to both comparisons. Following this, MetaboAnalyst 6.0 was employed to conduct correlation heatmap analyses, facilitating the evaluation of relationships among the differentially regulated metabolites. Following quercetin administration, a comprehensive analysis of metabolic pathways was conducted utilizing MetaboAnalyst 6.0 to pinpoint specific pathways showing alterations. The analysis entailed filtering lists of metabolites with upregulation and downregulation, based on a log base 10 ratio of peak intensity (log*p*) exceeding 2 and a pathway impact greater than 0.02. This filtering process ensured that the focus remained on the most significant changes in metabolic pathways, thereby minimizing the reporting of non-significant findings. Furthermore, MetaboAnalyst 6.0 was employed to identify key biomarkers within these pathways that were influenced by quercetin treatment. To address multiple testing issues, *p-*values were adjusted using the false discovery rate (FDR) method. The FDR approach is recognized as a conservative strategy for controlling the family-wise error rate in multiple testing scenarios, offering a more precise estimate of the true false discovery rate than traditional methods such as the Bonferroni correction.

It is important to note that the false discovery rate (FDR) method does not directly adjust *p-*values; rather, it provides a *q-*value for each test. A *q*-value indicates the expected proportion of false positives among the tests that surpass a specified significance threshold.

### 2.9 Integrated transcriptomic and metabolomic analysis

The integrated analysis of transcriptomic and metabolomic datasets was conducted utilizing the Metscape plugin within Cytoscape 3.7.0 to construct gene-metabolite interaction networks. This plugin facilitates the import of differentially expressed genes and differentially regulated metabolites into a network framework, enabling exploration of gene-metabolite associations and potentially elucidating the mechanisms underlying quercetin’s therapeutic effects on hepatic fibrosis. Specifically, Metscape imports these differentially expressed genes and metabolites into the network framework, automatically identifying edges that represent experimentally validated interactions between genes and metabolites. Furthermore, Metscape allows users to filter the network based on user-defined criteria and query it using predefined queries. For instance, Metscape allows the identification of sub-networks within the broader gene-metabolite interaction network that correspond to specific biological processes (BPs) or regulatory interactions. In this study, Metscape was employed to identify gene and metabolite sub-networks associated with specific biological processes and regulatory interactions, which may elucidate the therapeutic effects of quercetin on hepatic fibrosis. Specifically, Metscape facilitated the identification of sub-networks corresponding to biological processes and regulatory interactions, utilizing data from the KEGG database.^3^

### 2.10 Validation of RNA-Seq data by RT-qPCR

RT-qPCR validated selected DEGs to evaluate the reliability of the hepatic fibrosis model transcriptome sequencing data. Cpt1a, Acadm, Hadh, and Scd were chosen based on their close association with core pathways identified through transcriptomic analysis and KEGG enrichment analysis related to fatty acid *β*-oxidation, lipid metabolic regulation, and peroxisome proliferator-activated receptors (PPAR)-related signaling pathways. Cpt1a, Acadm, and Hadh play important roles in fatty acid β-oxidation, while Scd is involved in lipid synthesis and fatty acid desaturation. Thus, we selected these four genes as representative targets for validation of lipid metabolism-related RNA sequencing (RNA-seq) analysis. Gene expression levels were quantified using gene-specific primers with synthesized cDNA as the template, and β-actin (Actb) served as the internal reference gene for relative quantification. The primer sequences employed for RT-qPCR are detailed in Table 1. RT-qPCR was conducted using SYBR Premix Ex TaqTM (TaKaRa, Japan) on an Applied Biosystems 7500 Fast Real-Time PCR System (Thermo Fisher Scientific, USA).

**Table 1:** Primer sequences used for RT-qPCR validation.

Gene ID	Gene symbol	Forward primer (5′–3′)	Reverse primer (5′–3′)
12894	Cpt1a	CCTCCGTGGTGGAGAAGTAA	GGTGGAGTAGGCGACATTGA
11364	Acadm	TCCAGAGCAAGCACCTCAAC	GCCACATTCACCACCTTCAT
15107	Hadh	CAGCCTGGTCAAGAAGGTGT	TCCTGGTTCAGGGTCACTTC
20249	Scd1	CTGCTGGAGGTGCTGAATGT	AGGGCAGGTACTTGGTGGTA
11461	Actb	CCCGCGAGTACAACCTTCTT	CGTCATCCATGGCGAACTGG

The thermal cycling conditions comprised an initial denaturation at 95 °C for 10 min, followed by 40 cycles at 95 °C for 10 s and 60 °C for 60 s. Each experimental group consisted of three biological replicates, with each sample analyzed in triplicate as technical replicates. Relative gene expression levels were determined using the 2^−ΔΔCt^ method. Data are presented as mean ± standard error (SE). Statistical analyses were conducted using GraphPad Prism 6.0 software, with differences between groups assessed via Student’s *t*-test, and *p*-values indicating statistical significance.

### 2.11 Statistical analysis

IBM SPSS Statistics (version 26.0) was utilized for all statistical analyses. Experimental data were presented as mean ± standard deviation (SD) unless stated otherwise. The Shapiro–Wilk test assessed data distribution normality, while Levene’s test checked variance equality. For normally distributed data with equal variances, one-way analysis of variance (ANOVA) and Tukey’s test were applied to compare multiple groups. When variances were unequal, Welch’s ANOVA and Dunnett’s T3 test were used. A two-tailed *p* value of <0.05 indicated statistical significance. R statistical environment (version 4.3.0) was used for visualization and bioinformatics, with ggplot2 for graphing, dplyr for data processing, and biomaRt for gene annotation.

## 3 Results

### 3.1 Changes in inflammatory cytokines and fibrosis-related indicators in rat liver tissue

**Figure 1:**
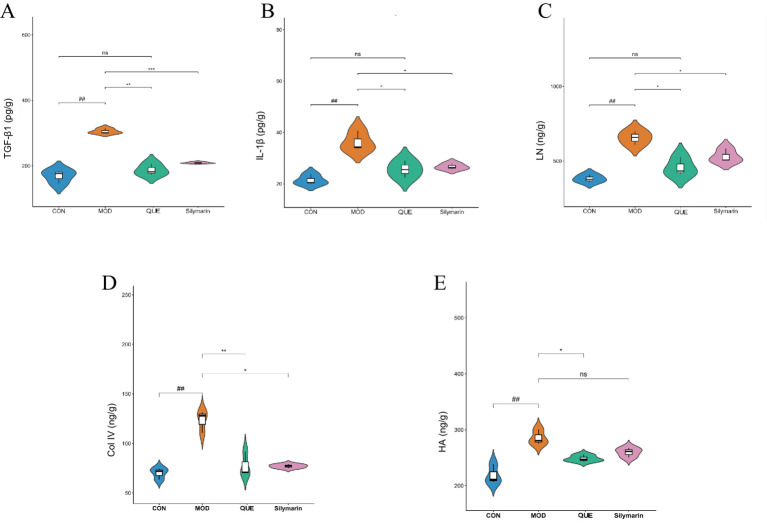
Changes in inflammatory cytokines and fibrosis-related indicators in rat liver tissue. **(A)** Hepatic TGF-β1 level; **(B)** hepatic IL-1β level; **(C)** hepatic LN level; **(D)** hepatic Col IV level; **(E)** hepatic HA level. Data are presented for each experimental group with *n* = 6 rats per group. CON, control group; MOD, model group; QUE, quercetin-treated group; Silymarin, silymarin-positive-control group. Compared with the CON group, ^#^*p* < 0.05, ^##^*p* < 0.01, ^###^*p* < 0.001; compared with the MOD group, *p < 0.05, **p < 0.01, ***p < 0.001; ns, not significant.

As illustrated in Table 1, the hepatic levels of TGF-β1, IL-1β, LN, Col IV, and HA were significantly elevated in the MOD group compared to the CON group (*p* < 0.001). Treatment with quercetin and silymarin resulted in a marked reduction in these hepatic markers in comparison to the MOD group (*p* < 0.001), with levels approaching normalization relative to the CON group (Figure 1). These findings confirm the successful induction of hepatic fibrosis via CCl₄ treatment. Additionally, quercetin treatment attenuated inflammation- and ECM-related markers when compared with the MOD group, indicating a reduction of fibrosis-associated inflammation and extracellular matrix production. These data validate our model for inducing hepatic fibrosis using CCl₄ treatment. The additional Silymarin group served as a positive control to demonstrate that the model was pharmacologically responsive. As doses between the silymarin and quercetin treatments were not dose equivalent, a direct comparison between treatments could not be concluded.

### 3.2 Histopathological changes in rat liver tissue

**Figure 2:**
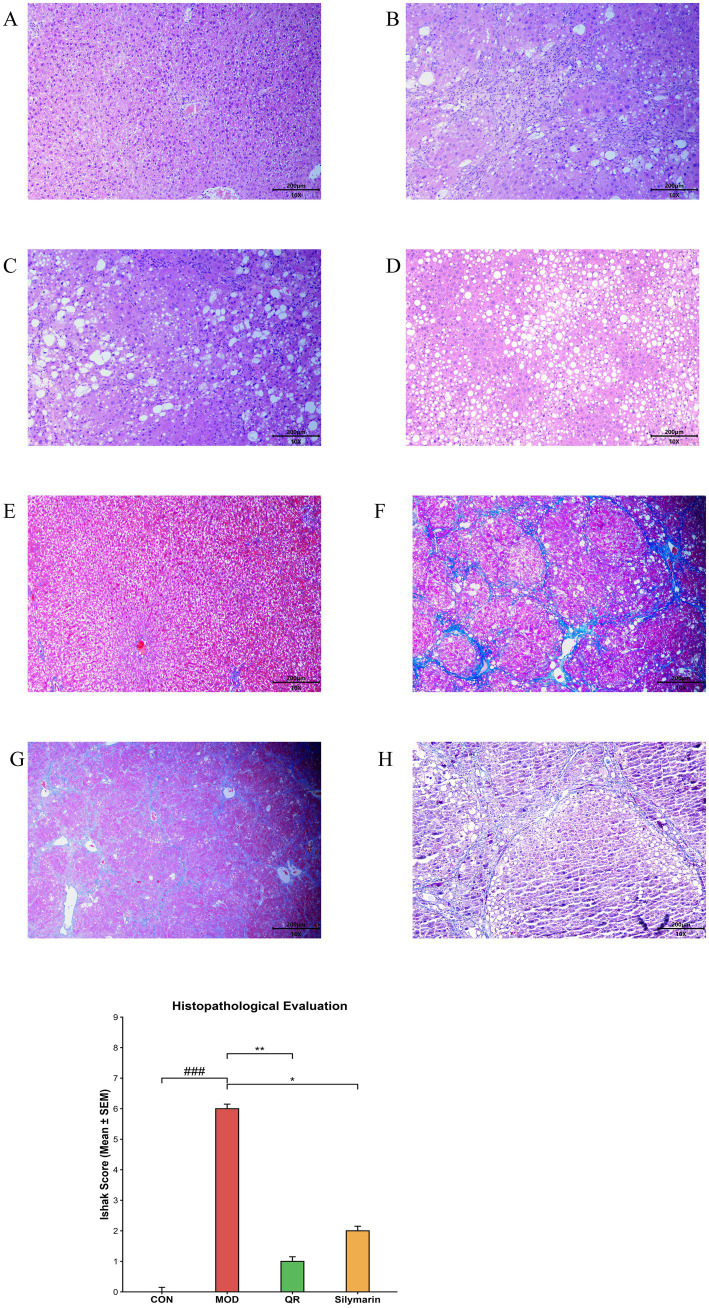
Histopathological evaluation of liver tissues in rats. Representative images are shown for each group, and histopathological scores were analyzed with *n* = 6 rats per group. **(A–D)** Representative hematoxylin and eosin (H&E)–stained sections of rat liver tissues; **(E–H)** Representative Masson’s trichrome–stained sections of rat liver tissues; **(I)** Histopathological scores of liver tissues assessed using the Ishak scoring system. CON, control group; MOD, model group; QR, quercetin-treated group; Silymarin, silymarin-positive-control group. Compared with the CON group, ^#^*p* < 0.05, ^##^*p* < 0.01, ^###^*p* < 0.001; compared with the MOD group, ^*^p < 0.05, ^**^p < 0.01, ^***^p < 0.001.

To evaluate histopathological alterations, hematoxylin and eosin (H&E)–stained liver sections from all experimental groups were analyzed for changes in both the overall organization of the liver tissue and individual cellular components. Liver sections from rats in the control (CON) group (refer to Figure 2A) exhibited normal hepatic lobules, characterized by a regular arrangement of hepatocytes and an absence of fatty degeneration or inflammatory cell infiltration. Conversely, liver sections from rats in the model (MOD) group (refer to Figure 2B) showed marked disruption of hepatic lobule architecture, accompanied by a significant infiltration of inflammatory cells into the liver tissue. Additionally, fat-laden hepatocytes (steatosis) and necrotic cells (necrosis) were observed. The Ishak score was quantified as described above and is shown in Figure 2I. The MOD group had significantly higher Ishak scores than the CON group, indicating successful induction of hepatic fibrosis (*p* < 0.001). Treatment with quercetin significantly decreased Ishak score compared with the MOD group, demonstrating partial reduction in fibrosis severity.

Remarkably, sections from rats administered either quercetin (refer to Figure 2C) or silymarin, the positive-control drug (refer to Figure 2D), exhibited predominantly normal liver lobules and a marked reduction in inflammatory cell infiltration and fatty degeneration within the liver tissue. Masson’s trichrome staining further corroborated the presence of normal hepatic lobular architecture in the control (CON) group, with an absence of blue staining of collagen fibers, which is indicative of fibrosis (refer to Figure 2E). In contrast, the model (MOD) group displayed substantial blue-staining collagen fibers in the portal areas and interlobular regions, forming a network of bridging fibrosis (refer to Figure 2F). Quercetin treatment (Figure 2G) or silymarin treatment (Figure 2H) resulted in attenuation of collagen fiber deposition within the liver tissue relative to the MOD group. Overall, histopathological changes support the successful induction of hepatic fibrosis within the rat model. Partial improvement of liver architecture was observed following quercetin treatment, together with reduced inflammatory infiltration and collagen deposition. Silymarin showed the expected positive protective histological trend and served as a positive-control reference group rather than a dose-equivalent comparator to quercetin.

### 3.3 Transcriptomic profiling of quercetin-treated rats with hepatic fibrosis

**Figure 3:**
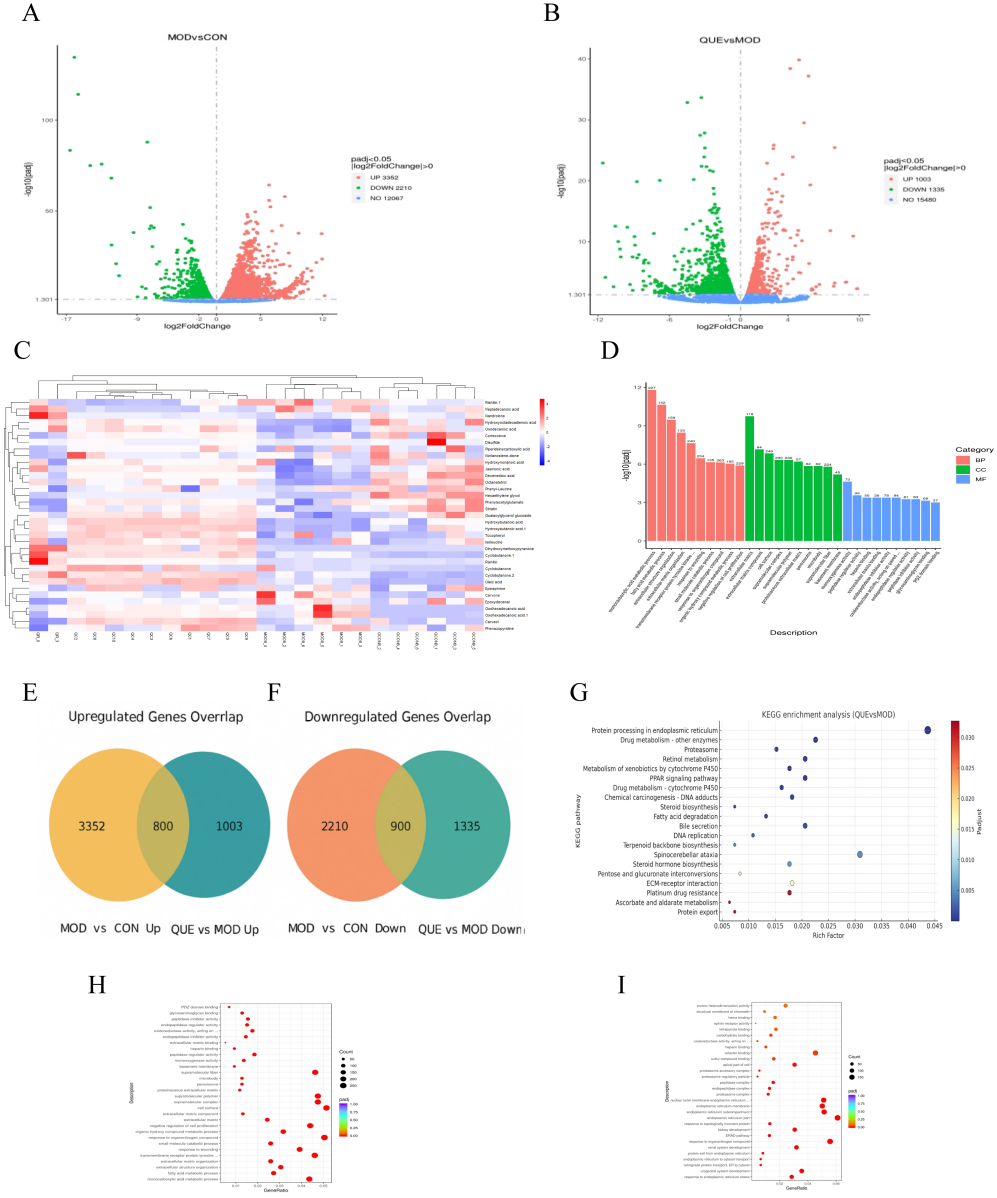
Transcriptomic profiling of quercetin (Que) treatment in rats with liver fibrosis. Transcriptomic analysis was performed on liver tissue samples from each group; the sample size per group was *n* = 6. **(A)** Volcano plot of differentially expressed genes (DEGs) between the control (CON) group and model (MOD) group. **(B)** Volcano plot of DEGs between the QUE-treated and MOD groups. **(C)** Hierarchical clustering heatmap of 35 core DEGs. **(D)** Gene Ontology (GO) functional classification of DEGs across biological process (BP), cellular component (CC), and molecular function (MF) categories. **(E–F)** Venn diagrams showing the overlap of commonly upregulated **(E)** and commonly downregulated **(F)** genes among groups. **(G)** KEGG pathway enrichment analysis of DEGs associated with QUE intervention. **(H)** GO enrichment bubble plot of core DEGs regulated by QUE. **(I)** Integrated refinement analysis of GO pathways illustrating pathway-level biological processes associated with quercetin treatment.

The findings suggest that quercetin treatment is associated with transcriptional changes in numerous genes implicated in CCl₄-induced hepatic fibrosis. To elucidate the impact of CCl₄-induced hepatic fibrosis on the comprehensive hepatic transcriptional landscape, we performed high-throughput RNA sequencing (RNA-seq) on liver tissues from control (CON) group, model (MOD) group, and quercetin-treated (QUE) group animals. The results, illustrated in Figure 3A through volcano plot analysis, reveal a substantial number of differentially expressed genes (DEGs) in the MOD group compared to the CON group. To be specific, 5,562 DEGs in the MOD group compared with the CON group were identified based on the screening criteria above. It is reasonable to expect that such a large set of DEGs might represent global changes in liver transcription due to chronic CCl₄ exposure, including inflammatory response, extracellular matrix remodeling, oxidative stress, and metabolic dysregulation. As such, the following pathway enrichment analyses were used to represent information at the pathway level and to identify trends rather than to suggest that each DEG has been validated as functioning as implied by the enrichment analysis. The majority of up-regulated DEGs were associated with extracellular matrix (ECM)-related proteins, such as COL1A1 and ACTA2, or lipid biosynthetic pathways. Conversely, most downregulated DEGs were linked to metabolic pathways, particularly fatty acid oxidation (FAO). These findings underscore that CCl₄ administration leads to extensive alterations in the liver’s transcriptomic profile, signifying substantial disruptions in both metabolic processes and ECM composition.

The impact of quercetin (Que) administration on the global hepatic transcriptional program following chronic CCl₄ exposure is depicted in Figure 3B. A comparative analysis between the QUE and MOD groups revealed a total of 2,338 differentially expressed genes (DEGs), with 1,003 genes exhibiting increased expression and 1,335 showing decreased expression. Venn diagrams (Figures 3E,F) highlight a subset of 1,700 DEGs that are altered under fibrotic conditions but demonstrate opposing expression trends following quercetin administration. Interestingly, several lipid metabolism-related genes (such as Cpt1a, Acadm, and Scd) were also differentially expressed in the QUE group relative to the MOD group. This implies quercetin treatment altered whole-liver expression patterns of lipid metabolism-related genes. However, because the transcriptomic data are from bulk liver tissue, they cannot determine whether these Cpt1a changes arose from hepatocytes, hepatic stellate cells (HSCs), or other cell populations within the liver.

Hierarchical clustering analysis (Figure 3C) reveals distinct global gene expression profiles across the MOD and CON groups, with the QUE group more closely aligning with the CON group. This suggests that quercetin treatment partially restored transcriptional profiles toward a non-fibrotic state, consistent with observed reductions in serum inflammatory markers (TGF-β1 and IL-1β) and ECM-related indicators (HA and COL IV) post-treatment. This indicates a coordinated molecular response to quercetin. KEGG pathway enrichment analysis of DEGs (Figure 3G) identified significant enrichment in pathways related to protein processing in the endoplasmic reticulum, the proteasome, and drug metabolism. Additionally, several genes annotated to PPAR-related lipid metabolism pathways exhibited expression patterns that were partially reversed following QUE treatment compared with the MOD group. In addition, several genes associated with retinol metabolism were found to be dysregulated in the MOD group, with partial normalization observed following quercetin treatment. This suggests a potential involvement of retinoid-related metabolic pathways in the progression and modulation of fibrosis.

We performed additional GO enrichment analysis to annotate transcriptomic changes functionally. As depicted in Figure 3D, GO functional classification terms of DEGs were enriched in 3 major categories. We displayed the GO enrichment bubble plot for core DEGs regulated by quercetin treatment (Figure 3H). The enriched terms were primarily related to oxidative stress responses, regulation of apoptosis, lipid metabolism, and extracellular matrix-related components. The summary plot (Figure 3I) of refined GO pathway analysis suggested unfolded protein response (UPR), endoplasmic reticulum stress response, lipid transport, and lipid oxidation-related processes might participate in the cellular response to quercetin. The enriched BP were predominantly associated with oxidative stress responses, regulation of apoptosis, and lipid metabolic processes. At the CC level, DEGs were primarily localized to the endoplasmic reticulum membrane, mitochondrial cristae, and structures related to the extracellular matrix. The molecular function (MF) analysis indicated enrichment in oxidoreductase activity, receptor binding, and enzyme regulatory activity. Further refinement of GO annotations highlighted significant enrichment of functional terms related to the unfolded protein response (UPR), endoplasmic reticulum stress, and lipid transport and oxidation. Collectively, the functional characteristics elucidated by the KEGG pathway analysis and GO analyses corroborate the hypothesis that quercetin influences the transcriptional regulation of protein homeostasis and energy metabolism in fibrotic liver tissue.

### 3.4 Serum metabolomics analysis

**Figure 4:**
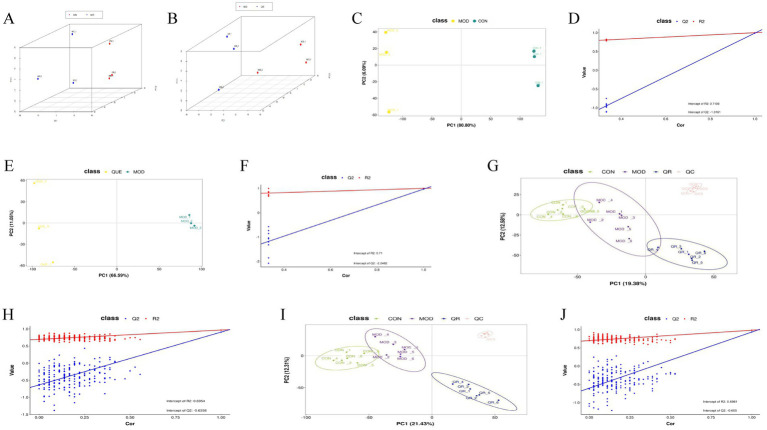
Metabolomic profiling of quercetin-treated rats with liver fibrosis. Serum metabolomic analysis was performed with *n* = 6 samples per group. **(A)** Three-dimensional PCA score plot comparing the control (CON) group and model (MOD) group. **(B)** Three-dimensional PCA score plot comparing the quercetin-treated (QUE) group and the MOD group. **(C)** Two-dimensional PCA score plot of the MOD and CON groups in the negative ion mode. **(D)** Permutation test of the OPLS-DA model for MOD vs. CON in the negative ion mode. **(E)** Two-dimensional PCA score plot of the QUE and MOD groups in the positive ion mode. **(F)** Permutation test of the OPLS-DA model for QUE vs. MOD in the positive ion mode. **(G)** PLS-DA score plot including CON, MOD, and QUE groups as well as quality-control (QC) samples in the negative ion mode. **(H)** Permutation test of the PLS-DA model in the negative ion mode. **(I)** PLS-DA score plot including CON, MOD, and QUE groups as well as QC samples in the positive ion mode. **(J)** Permutation test of the PLS-DA model in the positive ion mode. CON, control group; MOD, model group; QUE, quercetin-treated group (labeled as QR in some panels); QC, quality-control samples.

To assess the impact of quercetin (Que) intervention on the metabolic alterations in rats with CCl₄-induced liver fibrosis, we employed an untargeted serum metabolomics approach utilizing UPLC-QTOF-MS under both positive and negative ionization conditions. The raw mass spectrometry data were processed using Progenesis QI for metabolite identification and alignment, followed by multivariate statistical analysis conducted with EZinfo 3.0. In several figures presented below, the “QR” group denotes the cohort of rats administered quercetin (Que). PCA score plots of global metabolic differences between groups were done in three dimensions. PCA distribution between CON and MOD groups is shown in Figure 4A, where the metabolic profile was clearly shifted after exposure to CCl₄. The PCA distribution between the QUE and MOD groups is depicted in Figure 4B, which shows that treatment with quercetin partially reversed the fibrosis-associated metabolic profile. The negative ion-mode two-dimensional PCA score plot (Figure 4C) further demonstrated that the MOD group could be clearly separated from the CON group along PC1, which accounted for 80.80% of the variance. This finding indicates a significant alteration in the serum metabolic profile of the model group compared to the control group. In contrast, in the positive ion mode (Figure 4E), the QUE group exhibits partial overlap with the CON group while remaining distinct from the MOD group, suggesting a general normalization of the metabolic profile following quercetin treatment.

**Figure 5:**
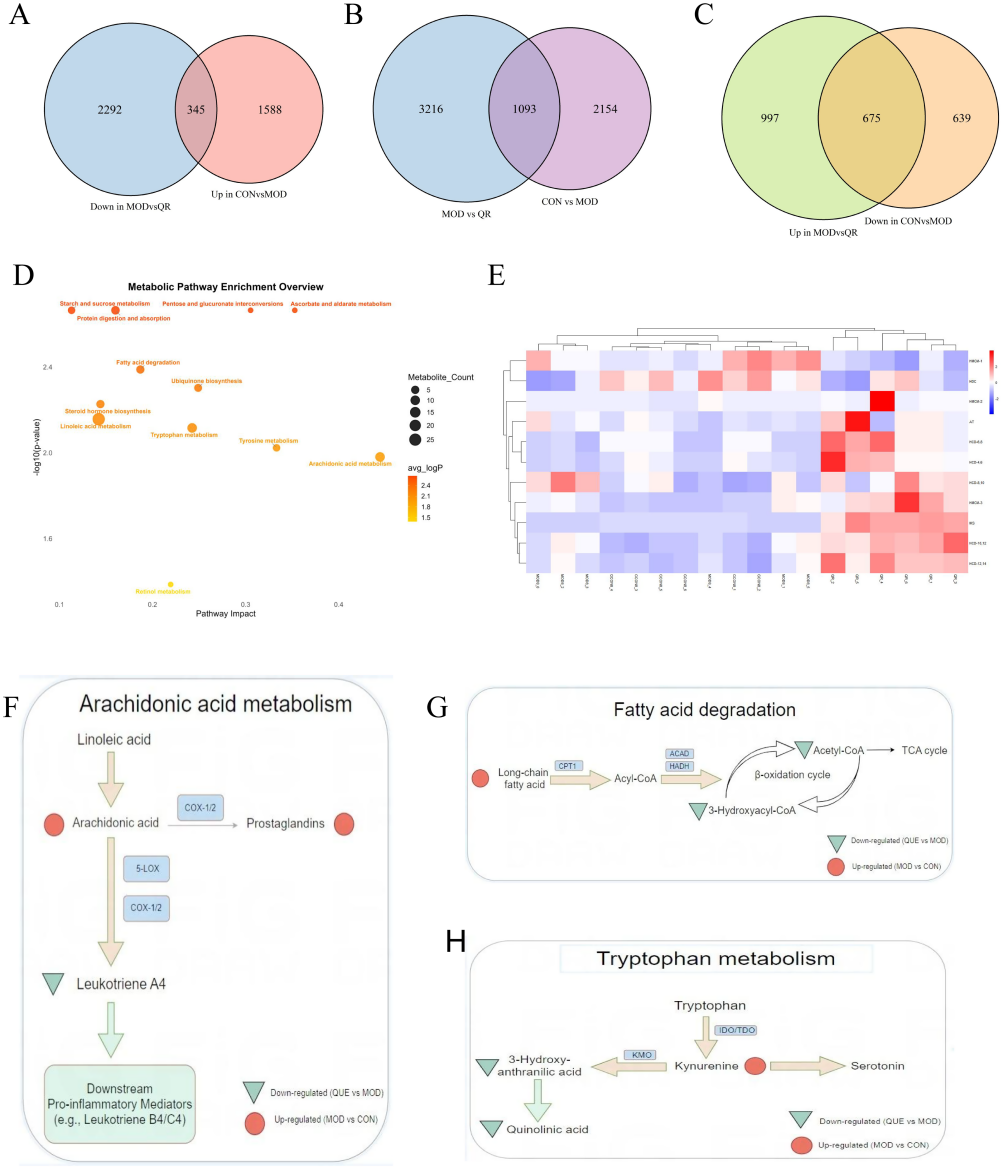
Metabolic pathway enrichment and inflammatory signaling analysis of quercetin (Que) intervention in rats with liver fibrosis. Differential metabolite screening and pathway enrichment were based on *n* = 6serum samples per group.**(A)** Venn diagram of differential metabolites between the control (CON) group and model (MOD) group. **(B)** Venn diagram showing the overlap between upregulated metabolites in CON vs. MOD and downregulated metabolites in MOD vs. QUE. **(C)** Venn diagram showing the overlap between downregulated metabolites in CON vs. MOD and upregulated metabolites in MOD vs. QUE. **(D)** Overview of enriched metabolic pathways. **(E)** Heatmap illustrating the expression patterns of differential metabolites across groups, with colors ranging from blue to red indicating normalized expression levels from low to high. **(F)** Schematic representation of alterations in the arachidonic acid metabolism pathway. **(G)** Schematic representation of alterations in the fatty acid degradation pathway. **(H)** Schematic representation of alterations in the tryptophan metabolism pathway. CON, control group; MOD, model group; QUE, quercetin-treated group. Red circles indicate upregulated metabolites (MOD group vs. CON group), whereas green triangles indicate downregulated metabolites (QUE group vs. MOD group).

The PCA score plots (Figures 4G,I) show tight clustering of QC samples, indicating the metabolomics platform’s analytical stability and reproducibility. To explore metabolic differences among groups, orthogonal projections to latent structures discriminant analysis (OPLS-DA) models were constructed and validated using 200-time permutation tests (Figures 4D,F). The OPLS-DA model comparing MOD and CON groups achieved *R*^2^*Y* = 0.94 and *Q*^2^ = 0.58 in negative ion mode, while the model for QUE and MOD groups in positive ion mode showed *R*^2^*Y* = 0.89 and *Q*^2^ = 0.45, indicating similar discrimination performance. Permutation test results confirmed that none of the permuted *Q*^2^ values exceeded those of the original models, suggesting that the observed group separation was not due to random chance and that there was no evidence of over-fitting. PLS-DA and permutation tests (Figures 4G–J) demonstrated a consistent separation between the groups, thereby reinforcing the reliability of the multivariate statistical methods employed. At the level of differential feature screening, Venn diagram analyses (Figures 5A–C) revealed a total of 2,292 differential metabolic features between the CON and MOD groups, suggesting a substantial reorganization of metabolism due to CCl₄-induced liver fibrosis. Following quercetin treatment, 1,020 core differential metabolic features (345 increased and 675 decreased) exhibited expression trends opposite to those observed in the MOD group, indicating a partial amelioration of fibrosis-related metabolic dysregulation with QUE treatment. Hierarchical clustering analysis (Figure 5E) further suggests that the overall metabolic profile of the QUE group more closely resembles that of the CON group than the MOD group. KEGG pathway enrichment analysis of the identified differential metabolites (Figure 5D) indicates that pathways such as fatty acid degradation, arachidonic acid metabolism, tryptophan metabolism, and linoleic acid metabolism are significantly enriched. These pathways are predominantly implicated in lipid metabolism, energy homeostasis, and inflammation-mediated biological processes. The pathway map visualizations (Figures 5F–H) depict the concurrent alteration of multiple nodes within metabolic pathways involved in the synthesis of inflammatory mediators, fatty acid metabolism, and amino acid metabolism following quercetin treatment. Collectively, these metabolic alterations imply that quercetin treatment is associated with the reorganization of several metabolic pathways in the liver fibrosis model.

### 3.5 Integrated analysis of transcriptomics and metabolomics

**Figure 6:**
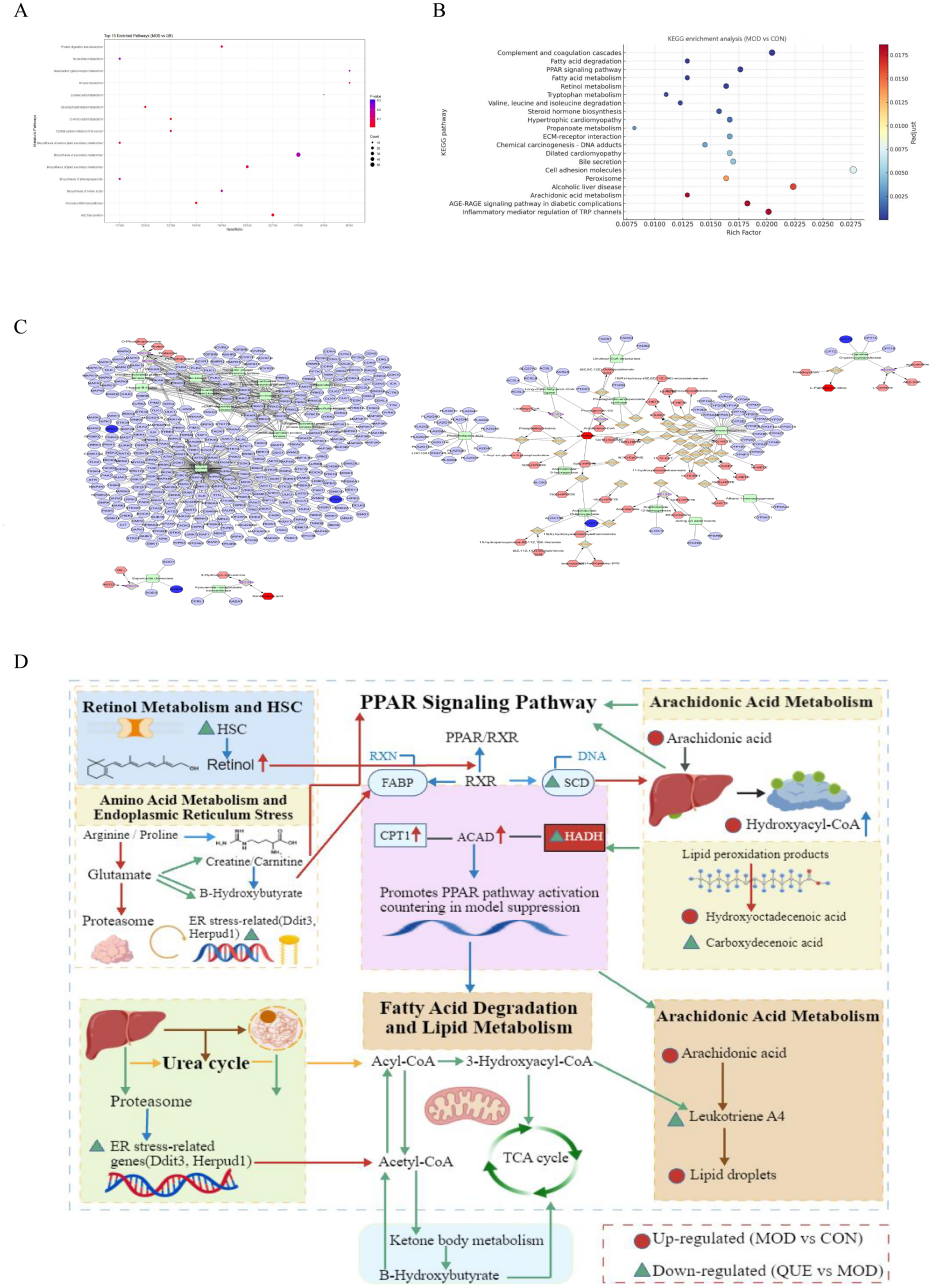
Integrated transcriptomic and metabolomic analysis of quercetin (Que) treatment in CCl₄-induced liver fibrosis in rats. Integrated analysis was based on transcriptomic and metabolomic datasets generated from *n* = 6 biological samples per group. **(A)** KEGG pathway enrichment bubble plot of differential metabolites between the MOD and QUE groups. **(B)** KEGG pathway enrichment analysis of differentially expressed genes (DEGs) between the MOD and CON groups. **(C)** Gene–metabolite interaction network constructed using Cytoscape with the Metscape plugin. **(D)** Schematic diagram illustrating the integrated multi-omics pathway-level associations between quercetin treatment and fibrosis-related transcriptomic and metabolomic alterations. In the schematic, colors indicate regulatory trends: red represents upregulation or restoration, and green represents downregulation. CON, control group; MOD, model group; QUE, quercetin-treated group.

The relationship between transcriptomic and metabolomic alterations induced by quercetin (Que) was investigated at a systems level through an integrated multi-omics approach, which combined differentially expressed genes (DEGs) and differentially metabolized features (DMFs). Initially, pathway enrichment analysis was conducted to identify pathways enriched in metabolomic data from both the MOD and QUE groups. The results of this analysis were visually represented in a bubble chart (Figure 6A). Numerous pathways related to amino acid metabolism, nucleotide metabolism, and lipid metabolism exhibited significant differences between the two groups, including protein digestion and absorption, nucleotide metabolism, and linoleic acid metabolism. These pathways demonstrated both high enrichment factors and metabolite counts, suggesting that quercetin treatment induces extensive, systemic modifications in fundamental metabolic pathways, akin to previously reported metabolic dysregulations associated with liver fibrosis.

In parallel, a KEGG pathway enrichment analysis was conducted to identify enriched pathways from the differentially expressed genes (DEGs) observed between the MOD and CON groups (Figure 6B). The analysis revealed that the enriched pathways were predominantly associated with complement and coagulation cascades, the PPAR signaling pathway, arachidonic acid metabolism, fatty acid degradation, and retinol metabolism. These results indicate significant alterations in inflammatory responses, lipid metabolic homeostasis, and energy metabolism within the liver fibrosis model, thereby establishing a transcriptomic framework for subsequent integrative analyses. To further elucidate the relationships between metabolic changes and transcriptional regulation, an integrated network analysis of differential metabolites and DEGs was performed using Cytoscape with Metscape (Figure 6C). The gene–metabolite interaction network demonstrated that key nodes were predominantly clustered in pathways related to lipid metabolism, amino acid metabolism, energy metabolism, and inflammation-associated processes. The metabolic pathways under investigation encompass arachidonic acid metabolism, fatty acid degradation, tryptophan metabolism, purine metabolism, retinol metabolism, arginine and proline metabolism, vitamin B6 metabolism, linoleic acid metabolism, oxidative phosphorylation, and sphingolipid metabolism. These pathways collectively form distinct yet interconnected modules within the network, illustrating the coordinated perturbations of multiple metabolic pathways in the liver fibrosis model and the subsequent reorganization of these pathways following quercetin intervention. Based on these findings, a comprehensive multi-omics schematic model was constructed to summarize pathway-level associations between quercetin treatment and fibrosis-related transcriptomic and metabolomic alterations. (refer to Figure 6D). This model incorporates key differentially expressed genes (DEGs), such as Cpt1a, Acadm, Hadh, and Scd, alongside differential metabolites, including arachidonic acid, hydroxyacyl-CoA, β-hydroxybutyrate, and leukotriene A4, within the context of the PPAR signaling pathway, arachidonic acid metabolism, fatty acid degradation, ketone body metabolism, endoplasmic reticulum stress, and retinol metabolism. In the model, red denotes upregulated or restored patterns (MOD group vs. CON group), while green indicates downregulated patterns following quercetin treatment (QUE group vs. MOD group). This overview illustrates the network-pathway relationships linking quercetin treatment to lipid–energy metabolism-related changes and fibrosis-related changes. They are not to be construed as direct proof that quercetin preserves hepatic stellate cell quiescence.

Furthermore, the integrated transcriptomic and metabolomic analyses reveal coordinated interactions among metabolic and transcriptional regulatory pathways in response to quercetin, particularly those involved in lipid metabolism, amino acid metabolism, energy metabolism, and stress-related processes. These multi-omics correlations offer a systematic framework for understanding the intricate molecular changes in the liver fibrosis model treated with quercetin. Nonetheless, the specific causal relationships and regulatory mechanisms necessitate further investigation through functional experiments targeting distinct aspects of the biochemical pathways.

### 3.6 Gene expression analysis by RT-qPCR

**Figure 7:**
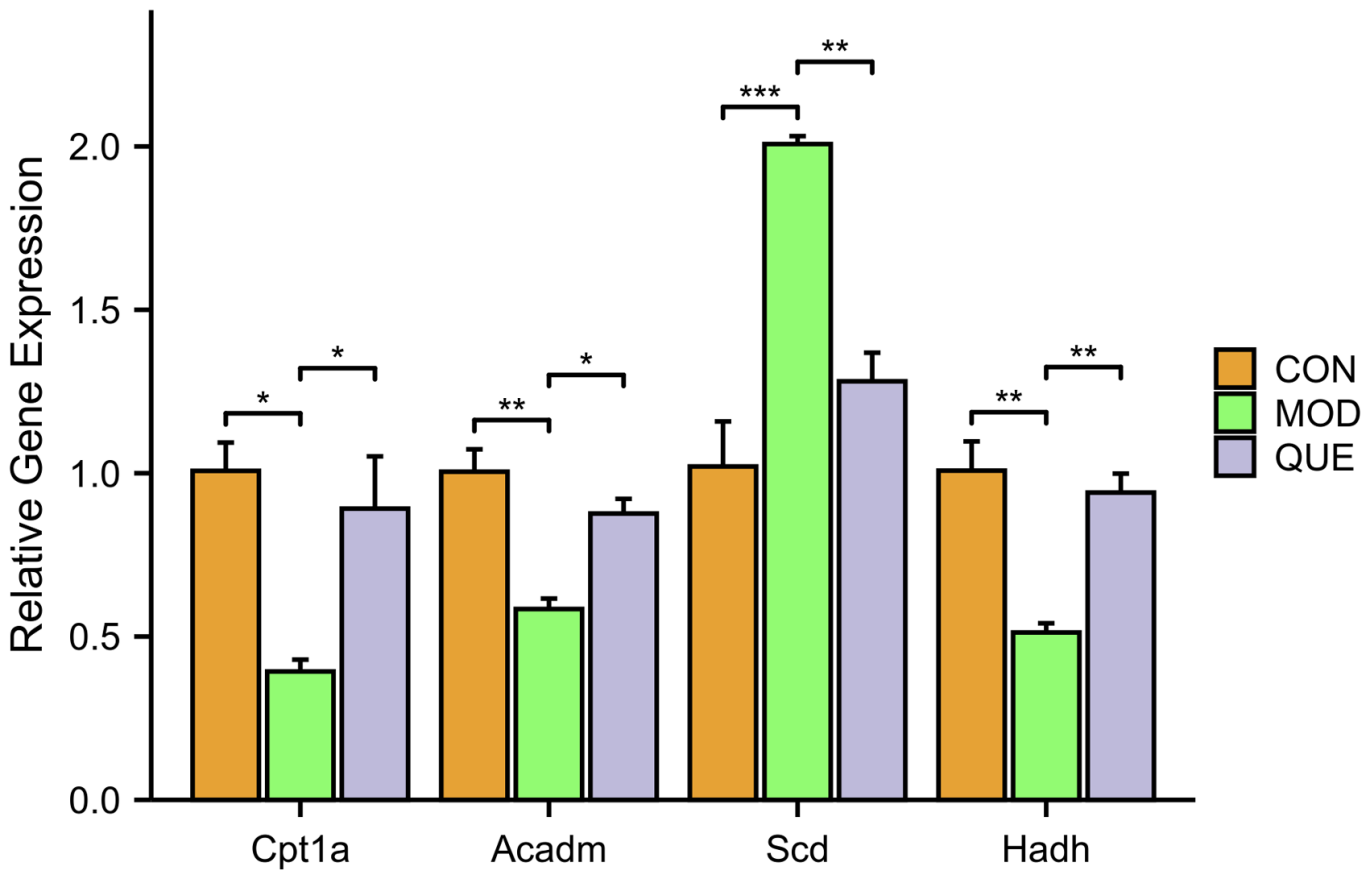
RT-qPCR validation of gene expression. Relative mRNA expression levels of Cpt1a, Acadm, Hadh, and Scd in CON, MOD, and QUE groups. Data were normalized to Actb and calculated using the 2^−ΔΔCt^ method. Values are presented as mean ± SE (*n* = 3 biological replicates per group). Compared with the CON group, ^#^*p* < 0.05, ^##^*p* < 0.01, ^###^*p* < 0.001; compared with the MOD group, *p < 0.05, **p < 0.01, ***p < 0.001.

Expression levels of selected lipid metabolism-related genes were confirmed with real-time quantitative polymerase chain reaction (RT-qPCR). Selected genes represented hub genes for fatty acid β-oxidation, lipid synthesis, and PPAR-related pathway enrichment from transcriptomic analysis. As depicted in Figure 7, the mRNA levels of Cpt1a, Acadm, and Hadh were significantly decreased, whereas the mRNA level of Scd was markedly increased in MOD rats compared to CON rats. These findings were consistent with the transcriptomic analysis, indicating that CCl₄-induced hepatic fibrosis is associated with reduced fatty acid β-oxidation and enhanced lipogenesis. Quercetin treatment significantly elevated the mRNA levels of Cpt1a, Acadm, and Hadh compared to MOD rats and decreased Scd mRNA expression. These findings indicate that treatment with quercetin results in at least partial reversal of fibrosis-associated alterations in whole-liver expression of lipid metabolism-related genes. The elevated whole-liver expression of Cpt1a should not be construed as evidence per se that quercetin exerts an antifibrotic effect on hepatic stellate cells because RT-qPCR performed on whole-liver tissue cannot determine cell-type-specific alterations in gene expression. Collectively, the RT-qPCR results corroborated the RNA-seq findings.

## 4 Discussion

Fibrosis, historically perceived as a benign wound-healing process characterized by inflammation and the accumulation of extracellular matrix (ECM), is now understood as an active disease phenotype influenced by multicellular interactions, metabolic reprogramming, and stress adaptation. Recent evidence indicates that hepatocytes, hepatic stellate cells (HSCs), and immune cells undergo significant transcriptional and metabolic alterations in response to chronic liver injury. Alterations in lipid metabolism, mitochondrial function, and nuclear receptor signaling have been implicated in the initiation, progression, and potential reversibility of fibrosis (22, 23). As such, investigating metabolic changes associated with fibrosis could shed light on mechanisms that are distinct from the classic anti-inflammatory approach. Nonetheless, due to CCl₄-induced fibrosis being predominantly driven by CYP2E1-mediated free-radical insult and hepatocyte injury, changes observed metabolically should be attributed to injury-induced changes instead of metabolic reprogramming driving fibrosis. One strength of this study is our coupling of global liver transcriptomics with RT-qPCR to demonstrate that QUE treatment was associated with alterations in fatty acid metabolism and PPAR-related pathways. Our RNA-seq analysis revealed significant downregulation of Cpt1a, Acadm, and Hadh at the transcript level, alongside significant upregulation of Scd in the CCl₄-induced fibrosis model. Subsequent RT-qPCR validation confirmed that QUE treatment effectively reversed these fibrosis-induced transcriptomic alterations. This strategy of “omics discovery coupled with targeted validation” challenges the notion that QUE possesses nonspecific anti-inflammatory or antioxidant properties, instead suggesting its role as a potential modulator of fibrosis-associated lipid metabolic reprogramming. Although these findings provide biologically plausible associations, they remain correlational and cannot establish direct mechanistic causality. Nevertheless, they provide a coherent list of targets for the future functional validation studies.

In particular, the genes that exhibited significant alterations included the simultaneous downregulation of Cpt1a, Acadm, and Hadh. CPT1A encodes a rate-limiting enzyme essential for the mitochondrial uptake of long-chain fatty acids for *β*-oxidation. In contrast, ACADM and HADH encode enzymes involved in subsequent stages of fatty acid oxidation (FAO). Considering their critical roles in FAO, we propose that the concurrent downregulation of these genes indicates a broader dysfunction in FAO rather than isolated enzymatic deficiencies. Empirical studies have demonstrated that impaired FAO results in lipid accumulation, lipotoxicity, and oxidative stress, which exacerbate inflammatory signaling and promote hepatic stellate cell (HSC) activation and extracellular matrix (ECM) production (22, 23). Consequently, the consistent downregulation of Cpt1a, Acadm, and Hadh in the model group is strongly associated with the metabolic disturbances characteristic of fibrosis. Furthermore, the restoration of these gene expressions following QUE treatment suggests its potential in mitigating fibrosis progression by reinstating fatty acid catabolism. This hypothesis is further supported by examining the impact of QUE on PPAR signaling pathways. Members of the PPAR family are integral in regulating hepatic fatty acid uptake, β-oxidation, and ketogenesis, with PPARα assuming a particularly significant role in these processes (24–26). While PPAR-signaling pathways clearly have translational significance in metabolic liver diseases like MASH/NASH, the model employed here was CCl₄-induced toxic fibrosis instead of metabolic disease. Thus, references to supporting data from PPAR-targeting drugs in MASH/NASH should be used to provide background support for the biological relevance of the lipid metabolism pathways discussed here, but should not be considered direct translational evidence for quercetin in CCl₄ fibrosis. Consequently, the QUE-mediated upregulation of Cpt1a, Acadm, and Hadh further illustrates that its antifibrotic properties may arise from the restoration of PPAR-dependent lipid metabolic processes. However, it is important to note that PPAR activity was not directly assessed, nor were receptor-specific inhibitors employed in this study. Thus, we do not claim that QUE directly activates PPARs; we propose that QUE treatment is implicated with the PPAR–fatty acid metabolism axis. Additionally, Cpt1a, Acadm, and Hadh are not solely regulated by PPARs. Transcriptional regulation of fatty acid oxidation-related genes may also be mediated by other transcription factors such as HNF4α and FOXA2. Given that PPAR transcriptional activity was not measured directly and receptor-specific antagonists were not employed, the involvement of PPAR signaling should be considered an associative pathway-level conclusion vs. a proven causal mechanism.

In contrast to other genes related to fatty acid oxidation, Scd was upregulated in the model group and subsequently downregulated following QUE treatment. SCD1, the rate-limiting enzyme responsible for converting saturated fatty acids into monounsaturated fatty acids (MUFAs), plays a critical role in lipogenesis, membrane remodeling, and lipid droplet formation (27). In hepatic stellate cell (HSC) models, the knockdown of stearoyl-CoA desaturase 1 (SCD1) has been pharmacologically demonstrated to reduce the expression of fibrogenic genes such as ACTA2 and COL1A1. This suggests that SCD1 may play a direct role in HSC activation and the fibrogenesis process (26). When considered alongside the aforementioned lipidomics data, the quercetin (Que)-induced downregulation of Scd suggests a shift away from a lipogenic state.

However, it is crucial to recognize that SCD1 expression may not exclusively promote a profibrotic function. Some studies suggest that hepatic SCD1 deficiency can lead to increased lipid saturation, which may activate inflammatory pathways and induce the expression of fibrosis-related genes under specific dietary or metabolic conditions (28–30). Thus, SCD1 might also serve a homeostatic function by protecting against the toxicity of saturated fatty acids and maintaining membrane fluidity (31–35). Consequently, while the QUE-induced downregulation of Scd in this study indicates a reduced need for lipogenesis following the restoration of lipid homeostasis, it should not be interpreted as definitive evidence that SCD1 is a profibrotic target.

It is important to acknowledge that increased CPT1A expression may not play the same role across all cell types. In this study, although Cpt1a expression was downregulated in the model group and subsequently returned to baseline levels following QUE treatment at the whole-liver level, genetic inhibition of CPT1A in hepatic stellate cells (HSCs) has been demonstrated to reduce HSC activation and fibrosis. This suggests that elevated fatty acid oxidation (FAO) within HSCs may enhance their fibrogenic activity (25). This phenomenon can likely be attributed to the differing metabolic requirements of hepatocytes compared to HSCs; hepatocytes utilize FAO to maintain lipid homeostasis and prevent lipotoxicity, whereas activated HSCs may exploit FAO as an energy source to support their proliferation and collagen synthesis (23, 25, 35). Thus, restoration of Cpt1a expression after QUE treatment should only be viewed as a whole-liver correlation with changes in transcriptional status related to fatty acid oxidation and not as evidence of a cell-autonomous antifibrotic role of Cpt1a in all liver cell types (36–40). This distinction matters because prior studies have shown that inhibition of CPT1A specifically in hepatic stellate cells may decrease HSC activation/fibrosis. For this reason, the current bulk RNA-seq and whole-liver RT-qPCR data cannot determine whether the change in Cpt1a occurred primarily in hepatocytes, activated HSCs, or other cell types within the liver. We thus refrain from making claims that restoration of Cpt1a mediates the antifibrotic effect of QUE. The future single-cell transcriptomic, spatial transcriptomic, or cell-specific functional experiments are needed.

QUE likely exerts its antifibrotic effects through a variety of interconnected mechanisms, beyond merely regulating metabolic gene expression. For instance, QUE has been demonstrated to directly inhibit TGF-*β*/Smad3 signaling and fibrogenic pathways in LX-2 cells, thereby reducing hepatic stellate cell (HSC) activation *in vitro* (27). Moreover, canonical fibrogenic pathways are probably not independent of metabolic processes, given the substantial crosstalk between inflammatory/injury signals and metabolic regulation. Inflammatory signaling pathways can impede nuclear receptor–dependent catabolic pathways, and restoring metabolic health may reduce inflammation and extracellular matrix (ECM) deposition. Consequently, we characterize QUE’s function as a network modulator of the “metabolism–inflammation–fibrosis” axis, rather than as a regulator of any single canonical pathway in a linear fashion.

We acknowledge several limitations inherent in our study. First, the use of RT-qPCR was constrained by a relatively small sample size, which reduced the statistical power to detect moderate effect sizes. Second, our data collection was limited to the transcript level, lacking insights from protein-level, enzymatic activity, or metabolic flux analyses. Second, only one dose of quercetin (50 mg/kg/day) was studied. As such, our study could not establish a dose–response relationship, identify the ideal efficacious dose or fully evaluate dose-dependent safety. Additional studies incorporating low-, medium- and high-dose quercetin groups should be performed to verify the therapeutic efficacy, safety, and pharmacological dose–response profile of quercetin treatment in hepatic fibrosis. It is worth mentioning that PPAR transcriptional activity was not directly assessed, nor were receptor-specific antagonists or genetic manipulations used. As such, alterations in Cpt1a, Acadm, Hadh, and Scd should be considered associations with PPAR-mediated transcription rather than markers of PPAR activation. Third, the CCl₄-induced fibrosis model employed may not accurately represent the pathogenesis or immune infiltration mechanisms associated with metabolic or cholestatic liver fibrosis etiologies. Lastly, the analysis of whole-liver tissues precludes the ability to determine cell-type specificity in the expression changes of Cpt1a and Scd. In addition, the metabolite screening criterion VIP ≥ 2 was relatively strict when compared with the widely used VIP > 1 threshold in metabolomics research. While this strategy improved metabolite selection specificity, biologically relevant metabolites with moderate discriminatory power might have been eliminated. Third, the interpretation of our dose setting of the positive-control drug should be cautious. The dose of silymarin/silybin we used was 200 mg/kg/day, higher than what has been commonly used in some preclinical research. Thus, the positive-control group mainly served as an internal control to verify that our CCl₄-induced fibrosis model responded appropriately, but not to establish a dose-equivalent comparison between silymarin and quercetin. The future research could administer multiple doses of silymarin (such as 50–100 mg/kg/day) and perform dose–response comparisons for rigorous evaluation of quercetin’s relative efficacy. Consequently, the future research should incorporate PPAR-dependent pharmacological and genetic interventions, protein-level validation, metabolic flux analyses, lipidomics, and cell-specific depletion studies to substantiate our findings.

## 5 Conclusion

Quercetin treatment concurred with reversal of CCl₄-induced hepatic fibrosis and alterations in whole-liver lipid metabolism-associated transcriptomic and metabolomic signatures. Whole-liver transcriptomic profiling and RT-qPCR validation revealed altered expression of fatty acid β-oxidation and lipid synthesis-related genes (i.e., Cpt1a, Acadm, Hadh, Scd). As transcriptomic and metabolomic analyses were conducted on whole-liver homogenates, the cell type driving these changes could not be identified. For this reason, Cpt1a upregulation should be considered an associative metabolic finding at the pathway level, rather than cell-specific evidence of an antifibrotic mechanism of action. The current study linked fibrosis to changes in lipid metabolism; however, because CCl₄ primarily induces liver injury through toxic free-radical-associated hepatocellular necrosis and not metabolically-driven fibrosis, these data do not suggest metabolic reprogramming as a causal contributor to fibrosis. Therefore, lipid metabolism- and NR-related pathways should be considered candidate mechanisms for the future studies.

In summary, the current study provides associative multi-omics evidence supporting an association between quercetin treatment and alterations in fibrosis-associated lipid metabolism in a CCl₄-induced hepatic fibrosis model. The future study incorporating a range of quercetin doses, cell-specific approaches, PPAR agonism assays, protein-level confirmation, and clinically relevant models of fibrosis is necessary to determine the validity of these findings.

## Data Availability

The raw data generated in this study can be found in the NCBI (https://www.ncbi.nlm.nih.gov/), accession PRJNA992181.

## Footnotes

1
https://www.omicstudio.cn/tool

2
https://www.omicstudio.cn/tool

3
https://www.genome.jp/kegg/
